# Long-term evaluation of coronary artery calcifications in kidney transplanted patients: a follow up of 5 years

**DOI:** 10.1038/s41598-019-43216-4

**Published:** 2019-05-03

**Authors:** C. Alfieri, L. Forzenigo, F. Tripodi, M. Meneghini, A. Regalia, D. Cresseri, P. Messa

**Affiliations:** 1 Unit of Nephrology, Dialysis and transplantation, Fondazione IRCCS Ca’ Granda Ospedale Maggiore Policlinico Milano, Milan, Italy; 2 Department of Radiology, Fondazione IRCCS Ca’ Granda Ospedale Maggiore Policlinico Milano, Milan, Italy; 3 Department of Clinical Sciences and Community Health, University of Milan, Milan, Italy

**Keywords:** Computed tomography, Kidney diseases

## Abstract

Coronary artery calcifications(CACs), are related to the increased cardiovascular mortality during kidney transplantation(KTx). Using coronary-CT performed at 1 month(T0) and 5 years(T5) after KTx we evaluated: (1) the prevalence of CACs; (2) the clinical and biochemical factors related to CACs; 3) the factors implicated with CACs progression. We evaluated 67-pts selected from the 103-pts transplanted in our unit between 2007 and 2008. Clinical and biochemical parameters were recorded at the time of pre-KTx evaluation and for five years after KTx. Coronary-CT for the Agatson score (AS) evaluation was performed at T0 and at T5, and CACs progression was determined. At baseline AS was 45 [0–233]. At T5 AS was 119 [1–413]. At T0, 69% of patients had CACs. Age and dialytic vintage were the main independent variables related to CACs. At T5, CACs were present in 76% of patients. Age was the only independent factor in determining CACs. A progression of CACs was observed in 74% of patients. They were older, had higher CACs-T0 and higher SBP throughout the 5-years. The presence of CACs at T0 and age were the only independent factors in determining the CACs-progression. CACs-T0 had the best discriminative power for CACs progression. CACs prevalence is quite high in KTx patients; Age is strictly related to CACs; Age and the presence of CACs at baseline were the two major factors associated with the progression of CACs during the five years of follow up. CACs-T0 had the best discriminative power for progression of CACs.

## Introduction

Patients affected by chronic kidney disease (CKD) have a relevant cardiovascular risk^1,2^. In those patients, in fact the prevalence and incidence of cardiovascular (CV) events are much higher than in the general population, and CV events are the most common cause of death. This fact can be related to the presence of many CKD specific CV risk factors that contribute to make CV risk stronger in those patients^3^.

In the stratification of CV risk, much importance is given to vascular calcifications. In particular, coronary artery calcifications (CACs) are considered to be a marker of coronary heart disease.

In clinical practice, CACs evaluation can be obtained using different methods such as Electron Beam tomography and by Multislice tomography. The two methods are similar in terms of sensitivity and specificity but, if the first one is characterized by high radiation exposition and is very expensive, the second one exposes patients to a lower quantity of radiation and is cheaper^4^.

In consideration of the potential high impact of CACs and of their relation with CV events and mortality, several clinical studies focused on the factors implicated on CACs progression have been realized^5,6^. In those studies, including mainly CKD and end stage renal disease (ESRD) patients, a higher rate of increase of CACs than in the general population and a relation among CACs progression, age and mineral metabolism factors have been reported^7–9^.

A small amount of data on the long term behavior of CACs in kidney transplantation (KTx) are available, and the few studies available are characterized by a small quantity of patients and a short observational time^10–12^.

Even if KTx reduces CV risk and mortality in CKD patients, in those patients CV risk remains higher than in the general population. So it might be important to calculate the impact of CACs in KTx and evaluate if the factors related with their evolution in the long term are similar to those reported in patients affected by CKD and end stage renal disease.

The aim of the present study is to explore, by means of Multislice Coronary CT (CT), the prevalence of CACs and the rate of their modification in a cohort of 67 patents evaluated for CACs at one month and at five years after KTx.

In addition, clinical and biochemical factors related with CACs progression will be investigated.

## Results

### Cohort characteristics

Our cohort of 67 patients was composed by 37 males and 30 females. Age of the whole cohort was 49 [39,57] years. The main clinical characteristics of our cohort are summarized in Table 1. Forty-five patients received hemodialysis before KTx and 59 patients were transplanted from a deceased donor. Only two patients had diabetes before KTx, whereas 22 patients underwent steroid therapy before KTx. Immunosuppressive therapy at T0, as reported in Table 2, was composed mainly of steroids, calcineurin inhibitors (CNI) and anti-proliferative drugs (MMF/MPA/AZA/mTOR-I).

**Table 1:** Clinical characteristics of the cohort studied.

Parameter	
Number of patients (n)	67
Type of dialysis n(%) (No/HD/PD)	5/45/17 (8/67/25)
Dialysis vintage (mths)	50 [36,70]
Age at Tx (years)	49 [39,57]
Gender (n) (M/F)	37/30
Kind of transplant n (%) (deceased donor/living donor)	59/8 (88/22)
Diabetes before KTx N (%)	2 (3)
Previous steroid therapy N (%)	22 (33)
CMV IgG pos N (%)	56 (84)
HCV pos N (%)	2 (3)
HbsAg pos N (%)	0 (0)

Footnotes: HD: Hemodialysis; PD: periotoneal diaysis; CMV pos: patients positive to cytomegalovirus; HCV pos: patients positive to virus of C Hepatitis; HbsAg pos: patients positive to HBV surface antigens.

**Table 2:** Ongoing immunosuppressive and antihypertensive at T0.

Drug	T0	T5
CyA/Tac/MMF-MPA/mTor inhibitor N (%)	4/63/64/3 (6/94/95/4)	4/44/56/10 (6/75/17/96)
Cumulative steroids during 1st yr of KTx (mg)	2715 [2605–2990]	NA
**Number of antihypertensive drugs (%)**		
0	12	8
1	39	25
2	22	42
3	22	17
>3	5	8

Footnotes: Cya: cyclosporine; Tac: Tacrolimus; MMF-MPA: mycophenolate; NA = not applicable.

Hypertension was mainly treated with one or two anti-hypertensive drugs. Beta blockers were the most used category of anti-hypertensive drugs (data not shown).

Clinical, blood and urinary parameters at the pre-KTx evaluation, at T0 and the average values T0-T5 are summarized in Table 3.

**Table 3:** Principal biochemical characteristics of our cohort at pre-Ktx evaluation at T0 and during the period T0–T5.

Parameters	Pre-KTx examination	T0	Average T0–T5	p*
SBP (mmHg)	NA	135 [120–150]	135 [124–143]	0.24
DBP ((mmHg)	NA	80 [80–90]	82 [77–85]	0.35
s-Creatinine (mg/dL)	8.0 [5.6–11.0]	1.41 [1.1–1.7]	1.5 [1.2–1.7]	0.09
Blood glucose (mg/dL)	91 [82–101]	74 [67–87]	79 [75–86]	**0.009**
Hb (g/dl)	11.3 [10.3–12.5]	10.8 [10–12]	12 [11–13]	**<0.0001**
PTH (ng/mL)	249 [148–470]	150 [94–217]	111 [80–179]	**0.01**
Ca (mg/dl)	9.2 [8.8–9.6]	9.8 [9–10]	9.8 [9.5–10.2]	0.28
P (mg/dL)	5.2 [4.3–5.7]	2.3 [2–2.8]	3 [2.7–3.4]	**<0.0001**
ALP (U/dL)	106 [62–171]	91 [68–114]	64 [54–78]	**0.01**
Prot-U (g/24 h)	NA	0.21 [0.15–0.33]	0.19 [0.15–0.32]	0.74
Total Cholesterol (mg/dl)	NA	226 [198–254]	211 [185–233]	**<0.0001**
HDL Cholesterol (mg/dl)	NA	61 [49–73]	57 [50–71]	**0.004**
Triglycerides (mg/dl)	NA	158 [119–209]	145 [118–191]	**<0.0001**

Footnotes: SBP: systolic blood pressure; DBP: diastolic blood pressure; PTH: parathormone- ALP: Alkaline Phosphatase; Bold format: statistical significance (p < 0.05).

During the five years of KTx, our patients didn’t show a significant decrease in renal function, whereas an increase in hemoglobin and serum phosphorus was found. In addition, we observed a significant reduction both of PTH and alkaline phosphatase levels.

### Coronary artery calcifications

In Table 4 all data concerning CACs evaluation at T0 and T5 are reported.

**Table 4:** Principal characteristics of CACs evaluations at T0 and T5.

Parameter	
AS-T0 (median [25%ile–75%ile) 45 [0–233]	p = **0.004**
AS-T5 (median [25%ile–75%ile) 119 [1–413]	
Categories of CACsT0 N (%)*	
No CACs	21 (31)
Small quantity	7 (10)
Slight quantity	8 (12)
Moderate quantity	22 (35)
High quantity	3 (4)
Very high quantity	6 (8)
Categories of CACsT5 N (%)*	
No CACs	16 (24)
Small quantity	6 (9)
Slight quantity	11 (16)
Moderate quantity	16 (25)
High quantity	11 (16)
Very high quantity	7 (10)
CACprog + N (%)	50 (74)

Footnotes: AS-T0: Agatson score at T0; AS-T5: Agatson score at T5; CACprog+: patients that had a progression of CACs using Sevrukov formula; CAC-cat+: Patients that increases their category of CACs. *CACs categories were defined according the indication presented in material and methods. Bold format: statistical significance (p < 0.05).

A significant increase of AS between the two evaluations has been observed. The mean value of AS, in fact, increased from 45 [0–233] at T0 to 119 [1–413] (p = 0.004) at T5.

### Coronary artery calcifications at T0

CACs were present in 69% of patients at baseline (CACsT0+). Clinical characteristics and pre-KTx biochemical exams are reported in Table 5. CACsT0+ were significantly older than patients without CACs at the same timepoint (CACsT0−) and had a longer dialytic vintage. No significant differences in pre-KTx biochemical status were found between the two groups. CACsT0+ had a worse control of blood pressure at T0, reflecting probably a worse vascular status than CACsT0-.

**Table 5:** Comparison between CAC-T0− and CAC-T0+.

Parameters	CACT0− (n = 21)	CACT0+ (n = 46)	p
Age (years)	42 [36–52]	53 [41–58]	**0.006**
Dialysis vintage (mths)	46 [33–50]	57 [39–76]	**0.03**
s-Creatinine (mg/dL)	9.2 [5.9–10.2]	10.1 [6.0–11.4]	0.46
Blood glucose (mg/dL)	87 [80–93]	92 [81–103]	0.34
Pre-KTx Hb (g/dl)	11.1 [10.2–12.0]	11.4 [10.4–12.5]	0.46
Pre-Ktx PTH (ng/mL)	310 [189–618]	279 [135–470]	0.14
Pre-Ktx Ca (mg/dl)	9.3 [8.8–9.6]	9.2 [8.9–9.7]	0.72
Pre-Ktx P (mg/dL)	5.5 [4.4–6.2]	5.3 [4.5–6.0]	0.87
Pre-Ktx ALP (U/dL)	90 [63–227]	105 [58–160]	0.99
SBP T0 (mmHg)	130 [117–135]	140 [120–150]	**0.03**
DBP T0 (mmHg)	85 [80–90]	80 [78–90]	0.59

Footnotes: SBP: systolic blood pressure; DBP: diastolic blood pressure; PTH: parathormone- ALP: Alkaline Phosphatase; Prot-U: urinary protein excretion; Bold format: statistical significance (p < 0.05).

In multivariate analysis, age at KTx and dialysis vintage were the main independent variables related both to presence and to the degree of CACs at T0 (Table 6).

**Table 6:** Multivariate analysis: Logistic regression for the event “CACT0+”.

Parameter	p	OR	IC	
Dialysis vintage (mths)	**0.004**	1.029	1.009	1.050
Age (yrs)	**0.0009**	1.090	0.036	1.146
SBP T0 (mmHg)	0.11	1.029	0.993	1.066

Footnotes: SBP: systolic blood pressure; Bold format: statistical significance (p < 0.05).

### Coronary artery calcifications at T5

At the evaluation performed at T5, CACs were present in 76% of patients (CACsT5+). Clinical characteristics and the biochemical differences between CACsT5+ and those patients without CACs at T5 (CACsT5−) are reported in Table 7.

**Table 7:** Biochemical differences between CACsT5− and CACsT5+.

Parameters	CACT5− (n = 16)	CACT5+ (n = 51)	p
Age (years)	37 [30–44]	53 [44–57]	**<0.0001**
Dialysis vintage (months)	48 [37–51]	53 [36–73]	0.52
SBP T0-T5 (mmHg)	124 [118–132]	133 [127–145]	**0.003**
DBP T0-T5 (mmHg)	81 [79–83]	82 [77–87]	0.42
s-Creatinine T0-T5 (mg/dL)	1.3 [1.2–1.7]	1.5 [1.3–1.7]	0.49
Blood glucose T0-T5 (mg/dL)	78 [73–81]	80 [76–90]	0.14
Hb T0-T5 (g/dL)	12 [11–14]	12 [11–12.8]	0.68
PTH T0-T5 (ng/mL)	119 [77–221]	111 [80–179]	0.80
Ca T0-T5 (mg/dl)	10 [9.6–10.3]	9.7 [9.4–10]	0.15
P (mg/dL) T0-T5	3.0 [2.7–3.4]	2.9 [2.6–3.4]	0.74
ALP T0-T5 (U/dL)	68 [52–79]	64 [56–78]	0.93
Total Cholesterol T0-T5 (mg/dl)	210 [187–231]	212 [184–238]	0.78
HDL Cholesterol T0-T5 (mg/dl)	58 [51–71]	56 [49–70]	0.74
Triglycerides T0-T5 (mg/dl)	130 [108–164]	148 [123–201]	**0.03**
Prot-U T0-T5 (g/24 h)	0.18 [0.15–0.22]	0.20 [0.15–0.32]	0.11

Footnotes: SBP: systolic blood pressure; DBP: diastolic blood pressure; ALP: Alkaline Phosphatase; Prot-U: urinary protein excretion; Bold format: statistical significance (p < 0.05).

CACsT5+ had higher AS at baseline: 130 [9.5–343] vs 0 [0–0.75], p < 0.0001. Moreover, they were significantly older. Differently from T0, no difference in dialytic vintage between the two groups was found. Blood pressure control was worse in patients with CACs at T5 during the 5 years of follow up.

In multivariate analysis (Table 8), age was the only independent factor in determining both degree and the presence of CACs at T5.

**Table 8:** Multivariate analysis: Logistic regression for the event “CAC-T5+”.

Parameter	p	OR	IC	
Dialysis vintage (mths)	0.72	1.004	0.984	1.024
Age (yrs)	**0.006**	1.130	1.035	1.234
SBP T0-T5 (mmHg)	0.48	1.030	0.948	1.120

Footnotes: SBP: systolic blood pressure; Bold format: statistical significance (p < 0.05).

### Coronary artery calcifications progression

**Figure 1:**
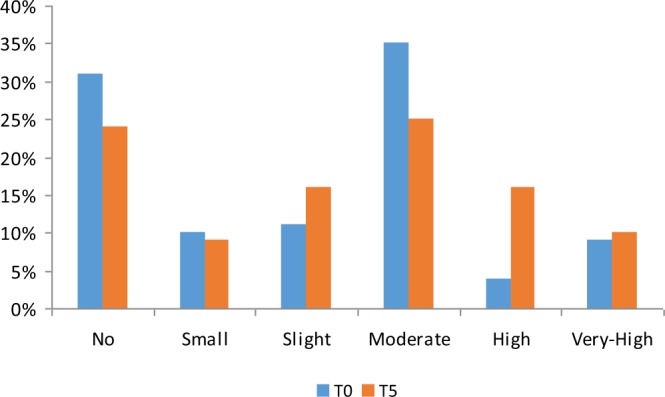
Distribution of the cohort (%) among the different categories of CACs at T0 and at T5.

As represented in Fig. 1, 31% and 24% of patients had no CACs at T0 and T5 respectively.

At baseline, 10% and 11% of patients were in small and slight CACs category, 35%, 4% and 9% of patients were respectively in moderate, high and very high category.

After five years, small and slight categories were assigned to 9% and 16% of patients whereas 25% 16% and 10% of patients had respectively moderate, high and very high CACs.

Figure 1 Using the method proposed by Sevrukov [16], a significant progression of CACs was observed in 74% of patients (CACprog+).

Compared to patients in whom no progression of CACs was observed (CACprog−), CACprog+ were older and characterized by a higher degree of CACs at baseline. In addition, they showed higher SBP during the period between T0 and T5. No other significant differences such as in dialytic vintage or in other biochemical parameters were found (Table 9).

**Table 9:** Differences between CACProg− and CACProg− +.

Parameters	CACProg− (n = 17)	CACProg+ (n = 50)	p
CACs-T0 (AS)	0 [0–2]	129 [9.4–265]	**<0.0001**
Age (years)	39 [30–46]	53 [43–57]	**0.001**
Dialysis vintage (months)	49 [40–60]	53 [36–72]	0.76
SBP T0-T5 (mmHg)	124 [118–133]	133 [127–145]	**0.01**
DBP T0-T5 (mmHg)	82 [79–84]	82 [77–88]	0.55
s-Creatinine T0-T5 (mg/dL)	1.4 [1.2–1.7]	1.5 [1.3–1.7]	0.47
Blood glucose T0-T5 (mg/dL)	79 [74–82]	79 [75–88]	0.33
Hb T0-T5 (g/dL)	12 [11–14]	12 [11–13]	0.77
PTH T0-T5 (ng/mL)	103 [77–202]	79 [75–88]	0.33
Ca T0-T5 (mg/dl)	10 [9.6–10.3]	9.7 [9.4–10]	0.16
P (mg/dL) T0-T5	3 [2.7–3.5]	3 [2.7–3.3]	0.49
ALP T0-T5 (U/dL)	70 [52–81]	64 [56–78]	0.62
Total Cholesterol T0-T5 (mg/dl)	221 [179–235]	211 [190–228]	0.86
HDL Cholesterol T0-T5 (mg/dl)	57 [47–72]	57 [50–70]	0.90
Triglycerides T0-T5 (mg/dl)	131 [97–172]	147 [121–194]	0.20
Prot-U T0-T5 (g/24 h)	0.18 [0.15–0.23]	0.19 [0.15–0.34]	0.15

Footnotes: SBP: systolic blood pressure; DBP: diastolic blood pressure; ALP: Alkaline Phosphatase.

Prot-U: urinary protein excretion; Bold format: statistical significance (p < 0.05).

The impact of immunosuppressive therapy on CAC progression was investigated, and a possible influence of calcineurin inhibitors treatment, in particular Tacrolimus, was evidenced. No other differences were found in the rest of immunosuppressive therapy and in anti-hypertensive drugs (Table 10).

**Table 10:** Differences between CACProg− and CACProg− + in therapy.

Parameters	CAC-Prog− (n = 17)	CAC-Prog+ (n = 50)	p
Gender (n) (M/F)	7/10	20/30	0.17
Type of dialysis n (%) (No/HD/PD)	1/12/4 (7/70/23)	12/34/4 (24/68/8)	0.95
Kind of transplant n (%) (deceased donor/living donor)	14/3 (82/12)	45/5 (90/10)	0.40
Calcineurin Inhibitors therapy T5 n (%) (No/Cya/FK)	1/3/13 (7/17/76)	10/1/39 (20/2/78)	**0.04**
Steroid Therapy T5 n (%) (No/Yes)	1/16 (6/94)	1/49 (2/98)	0.42
mTor-i therapy T5 n (%) (No/Yes)	16/1 (94/6)	39/11 (78/22)	0.20
MMF therapy T5 n (%) (No/Yes)	0/17 (0/100)	3/47 (6/94)	0.39
Number of antihypertensive drugs (%)			
0	5	8	0.69
1	36	18	
2	43	38	
3	11	28	
>3	5	8	

In multivariate analysis, the presence of CACs at T0 and the age were the only independent factors in determining the CAC progression (Table 11).

**Table 11:** Multivariate analysis: Logistic regression for the event “CAC-Prog+”.

Parameter	p	OR	IC	
CACs-T0+	**0.0007**	20.5	3.55	118.2
Dialysis vintage (mths)	0.19	0.984	0.960	1.008
Age (yrs)	**0.05**	1.093	1.0	1.19
SBP T0-T5 (mmHg)	0.75	0.98	0.90	1.07

Footnotes: SBP: systolic blood pressure; Bold format: statistical significance (p < 0.05).

**Figure 2:**
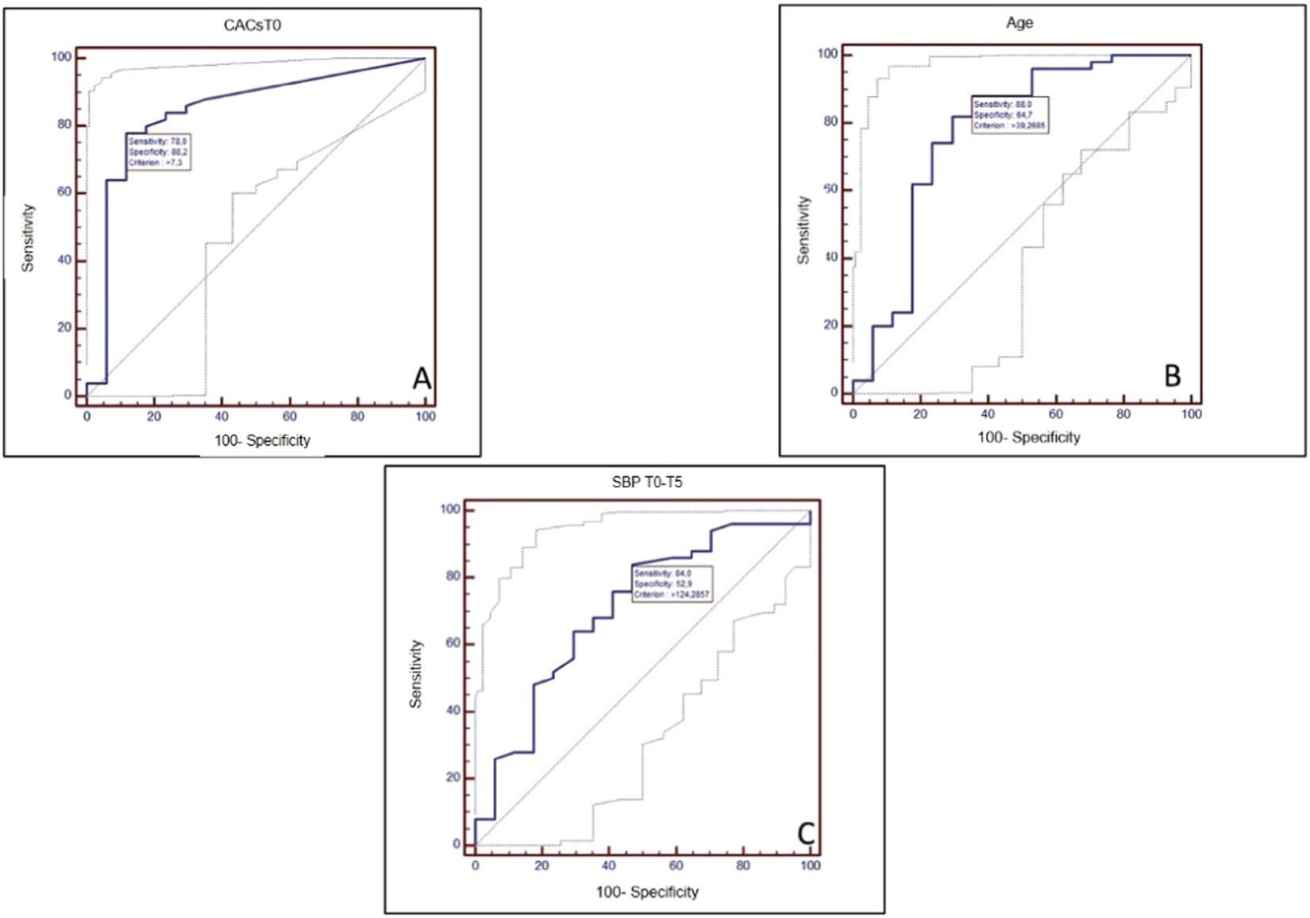
ROC curves for the event “CAC-Prog+”. (**A**) Dependent variable- CACsT0. (**B**) Dependent variable- Age at KTx. (**C**) Dependent variable- SBP T0-T5.

To evaluate the discriminatory power of the three most related parameters (CACs-T0+, age and T0-T5 SBP) in identifying those patients affected by a higher risk of CACs progression, ROC curves analyses were performed (Fig. 2). They demonstrated the good predictive value of CACsT0 (AUC: 0.84 ± 0.06, p < 0.001), age (AUC = 0.77 ± 0.07, p = 0.001) and T0-T5 SBP (AUC = 0.70 ± 0.07, p = 0.01) for identifying CACProg+ patients.

Interestingly, the insertion in the same model of CACsT0 and age considered together was followed by a reduction of 7% of the AUC respect the model including only CACsT0, increasing the importance of CACsT0 *per se* (without additional factors) in CACs progression. From ROC analysis, the best cut-off CACsT0 AS value able to identify those patients that will increase significantly CACs was 8.3 AS (sensibility: 78.0% - specificity: 88.2%).

### Cardiovascular events and death

During the five years of follow up, ten patients (four in the first year of KTx) had a cardiovascular event (CV+). In four patients heart event (i.e.: ischemic attack- arrhythmia) occurred. Interestingly, all of them had a moderate/high degree of CACs both at T0 and T5- and were CACprog+ (p < 0.0001). No death nor graft loss have been reported.

## Discussion

The first aim of our study was to evaluate in a cohort of KTx patients the prevalence of CACs at one month and after five years of transplantation. At T0, 69% of the cohort presented CACs and most of CAC-T0+ were within the category with moderate amounts of coronary calcium. These results are substantially in line with those reported in the literature, where a prevalence of CACs at the moment of transplantation between 35% and 70% is reported^13,14^.

In 31% of patients no CACs at baseline were found.

In our study, the correlations between CACs-T0 and some parameters before KTx have been explored. In agreement to the data reported in the literature, age and dialysis vintage were strongly related to the presence and the degree of baseline CACs^15^. In clinical trials, also performed by our Group, the relationship among age, dialysis vintage and vascular calcifications has been demonstrated also in other sites, as for example in abdominal aorta^16–18^. Interestingly, no correlations between pre-KTx biochemical parameters and CACs-T0 have been found. Some studies have also hypothesized that genetic factors might have an important role in the promotion of CACs development and progression both in dialysis time and after KTx^19^.

The correlation demonstrated between CACs and SBP at T0 probably reflects a worse cardiovascular condition of CACT0+, and therefore might be considered as a consequence, more than a determining factor of the calcification process.

Most of the correlations found at T0 were also confirmed at T5, when 76% of the patients had CACs. The subdivision into categories according to AS, evidenced an increase of the prevalence of CACs of moderate-high degree category from T0 to T5 evaluation. Also at T5 a correlation of age and SBP average levels with the presence and quantity of CACs was demonstrated. Interestingly, at T5 the correlation with the dialysis vintage was not confirmed, and dialysis vintage was not different between CAC-Prog− and CAC-Prog+. This might indicate the presence of other factors implicated in the promotion of the calcification process during the life of the transplant.

In addition, it is important to underscore the direct correlation between CAC-T0 and CAC-T5. This probably reflects the inability of the KTx condition to reduce CACs, but rather to slow their progression respect to the dialysis status^20^. In the literature there are numerous data, frequently contradictory, concerning the effect of KTx on the progression of the CACs. Among them, the study of Adamidis *et al*. compares 20 KTx patients previously treated with hemodialysis with a group of 16 dialysis patients still in control. The basic evaluation shows a high prevalence of CACs in both groups. Also in this study close correlation between CACs and the patient’s age was described. In the follow-up (mean of 16 months) a slower progression of the calcific process in the KTx group was documented^21^. In another study, conducted on 281 patients, Marechal *et al*. were not able to demonstrate a benefit of the transplantation on coronary calcification. The main results, in fact, report that the progression is present mainly within the first four years of transplantation and that the baseline quantity of calcifications is an independent determinant of CACs progression^xx^.

The second aim of our study was to evaluate during follow-up the prevalence of CACs progression and to try to identify the factors related with this event. As in other studies, for the identification of patients in which the amount of CACs increased, we used the formula proposed by Sevrukov^22^. The progression of CACs was observed in 74% of patients, with an increase of the percentage of subjects who fell into the categories with the highest AS. Again, also CACs progression was significantly correlated to age, to a higher Agatston score at T0 as well as to a higher average SBP during the five years between the TC evaluations. Interestingly, no significant correlations were found with the average values of biochemical parameters obtained in the five years, including mineral metabolism parameters, and this allows us to assume that once the calcification process began, self-maintained processes, irrespective of metabolic-mineral status, might be present. However, the lack of these correlations could also be attributed to the limited period of follow-up, although five years is a fairly long time compared to many studies in the literature.

In 2009, Abedi *et al*. explored the effect of renal transplantation on the calcium scores of coronary arteries among 31 hemodialysis patients. They demonstrated a significant reduction of CACs after six months of KTx respect pre-KTx evaluation. In addition, a significant linear correlation between AS and iPTH and Ca-P product reduction was found^11^. These results might seem to be partially in contrast with ours. It is important however to underscore that some methodological differences are present between the two studies. In our study, that included a higher number of cases, the second CACs evaluation has been performed significantly later after KTx (five years). In addition, CACs progression was defined differently in the two studies and by means of two different methods. As in Abedi *et al*. study, according to our results CAC-Prog + patients showed a tendency (not statistically significant after the application of not-parametrical tests) to have lower levels of PTH. Of note, in our study, considering the high variability of PTH levels, the T0-T5 average levels, and not the single PTH level at the moment of the second evaluation, have been considered. The same was done for the other parameters of mineral metabolism.

With the aim to explore the discriminating power for CACs progression of the three most related parameters, ROC analysis was performed. ROC analysis confirmed the good correlations already evidenced for all the three parameters, and identified the presence of CACs at T0 as the strongest discriminating factor for CACs progression, with an AUC of 0.84 and a threshold value for CACsT0 AS of 8.4.

During the five years of follow-up, 10 of our patients developed cardiovascular events. All these patients were both at T0 and T5 in moderate/high degree categories, as well as in the group of subjects in which CAC progression was observed. Even if obtained in a small cohort of patients, this result, associated to those evidenced by ROC curves, could stress the importance of the accurate cardiovascular study before KTx and of the regular cardiovascular follow up to identify those patients at higher cardiovascular risk.

The principal limitation of our study is the small number of patients, which does not allow a proper assessment of the different distribution of patients within the categories of AS and does not give the opportunity to evaluate deeply the factors related to the change of category.

In consideration of the main purposes of our work, we can conclude that in our cohort of KTx patients:

- the prevalence of CACs at the time of transplantation is quite high;
- that prevalence is also confirmed in the subsequent assessment performed five years after kidney transplantation, in which an increase in patients with coronary calcifications was demonstrated;
- there is a strict relation between CACs and age, both at T0 and T5;
- Age and the presence of CACs at baseline are the two major factors associated with the progression of coronary calcifications during the five years of follow up, and among them the amount of CACs at T0 was found to have the best discriminative power among patients with progression of coronary calcifications.

The results obtained in our study validate the need to consider, in specific cases, the amount of coronary calcium in pre-transplant evaluation of patients on dialysis.

This approach could be useful to identify those patients at increased cardiovascular risk and to set up a comprehensive evaluation in the post-transplant time.

## Material and Methods

### Cohort characteristics

A total of 67 patients (M = 37), randomly selected from the 103 patients transplanted in our Unit between January 2007 and December 2008 have been examined. Clinical and biochemical characteristics of studied patients at baseline were not different to those of patients excluded from the study.

At the first month (T0) and after five years of KTx (T5), each patient underwent a thoracic tomography for the evaluation of CACs.

After KTx all patients were followed according to routine clinical indications of our Department.

The protocol was approved by the Ethics Committee of Fondazione IRCCS Ca’Granda Ospedale Maggiore Policlinico of Milan, and was conducted according to the ethical principles of the Helsinki Convention, and each patient signed an informed consent. No organs/tissues analysed for this study were procured from prisoners.

### Biochemical evaluations

Biological samples were collected in our Department from each patient after 12 hours of fasting, and all biochemical analyses were performed in the same laboratory at our Institution. Biochemical data observed at the moment of the pre-transplant examination and at T0 were recorded. To take into account the global exposition of patients during the five years of follow up, between T0 and T5 clinical and biochemical parameters were recorded, and considered in statistical analysis as average values.

Jaffe method was used to dose serum creatinine, whereas daily urinary protein excretion (Prot-U) was determined by immunoturbidimetric method.

Parathormone (PTH) levels were quantified using ECLIA (ImmunoAssay in ElettroChemiLuminescent) method by Roche by means of modular analytics E170. The Measure range was 1.20–5000 pg/mL The Conversion was: pg/mLx0.106 = pmol/L. Normal range: 15–65 pg/ml.

All the other biochemical parameters were evaluated according to routine methodology used in our central laboratory.

### Computed tomography and Agatson Calcium Score quantification

Radiological images were acquired with a scanner Somaton Definition Dual Source 64 slices.

Briefly, the patient, supine on the tomography table was prepared to be ECG monitored. The optimal cardiac frequency for a good acquisition of the images was set at 50–60 bpm. The sequential acquisition of default was performed automatically by the scanner during the interval ECG R-R, to evaluate images only in one precise phase of the cardiac cycle. The radiant dose to which patients were exposed was around 1.5–2 mSvs, more contained in comparison to the examinations with retrospective gating (4–7 mSvs)^23^.

**Figure 3:**
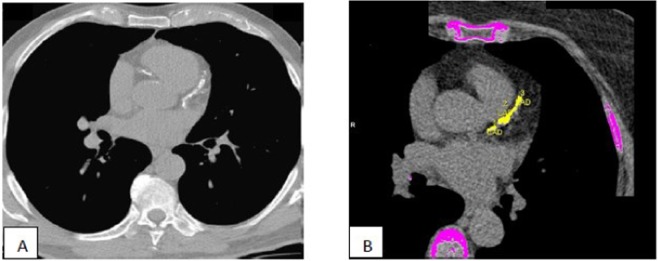
Calcification of anterior descendent coronary artery detected using TC (**A**). Coronary arterial calcifications evaluated using Agatson score (**B**).

After the scan, the acquired images were re-elaborated, using specific software Siemens, for the measurement of the CACs according to the method of Agatson^24^. In particular, the software used identifies all the pixels with density> 130 HUs and attributes them a code color on the next elaborated images. The radiologist identifies the center of the coronary calcification attributing a different color according to the center of the calcific plate, distinguishing common coronary, descending anterior, circumflex, right coronary, and descending back coronary (Fig. 3).

According to Agatson method, the software multiplies the ampleness of the identified area of at least 0.52 mm^2^ (2 pixels) with density >130 UH for one of the following factors^25^:

- 1 for values from 130 to 199 UH;
- 2 for values from 200 up to 299 UH;
- 3 for values from 300 to 399 UH;
- 4 for values >400 UH;
  The total value of the calcification is obtained by the sum of the scores of the single areas identified in every coronary artery applying her two specific equations for Agatson score (AS) and volumetric score (VS).
  These measurements are effected on the whole tree coronary and they allow to distinguish four degrees of gravity for a progressive increase of the quantity coronary calcium:
- Score 0–1, absence of CACs
- Score 1–10, small quantity;
- Score 11–100, slight quantity;
- Score 101–400, moderate quantity;
- Score 401–1000, high quantity
- Score >1000, very high quantity.

The method proposed by Sevrukov for CACs progression determination was used. According to this method, in presence of two different evaluations of CACs:

- in subjects with detectable CACs at baseline, the smallest statistically significant interval change indicative of CACs progression is defined by the formula (4.93 ×√baseline CACs)
- in subjects with a baseline CACs = 0, a follow-up CACs >11.6 indicates progression^26^.

### Definition and data collection of cardiovascular events

The following cardiovascular events have been considered for this paper: ischemic heart attacks (STEMI and N-STEMI), arrhythmias, brain ischemia and hemorrhages. All the cardiovascular events were reported in written clinical records by the transplant medical doctors of our Unit.

### Statistical analysis

In statistical analysis, continuous variables were expressed as median value [25%ile; 75%ile] and were log transformed if they had a skewed distribution.

Differences among groups were determined by Student’s t test, Mann-Whitney, Kruskal-Wallis test and ANOVA, where indicated.

Differences among percentages were determined by χ^2^ or Fisher test.

Linear regression and logistic regression were employed in order to perform uni-variated and multi-variated analysis respectively.

Clinical and biochemical parameters were considered as the value recorded at the moment of the first CAC evaluation (T0) and the average values recorded during the five years of follow up (T0-T5).

Patients with AS <1 were considered as not affected by CACs.

The discriminating power of different parameters for identify CACProg+ patients was analysed by means of Receiver Operating Characteristic (ROC) curves.

Statistical analysis was performed using software Statistica^®^ version 10 and SPSS version 20^®^ and significance was set for p values < 0.05.

### Other statements

We declare that no organs/tissues analysed for this study were procured from prisoners. If needed, data are available in anonymous form.

### Ethical approval

All procedures performed in studies involving human participants were in accordance with the ethical standards of the institutional and/or national research committee and with the 1964 Helsinki declaration and its later amendments or comparable ethical standards.

### Informed consent

Informed consent was obtained from all individual participants included in the study.
